# CP and CP-PGN protect mice against MRSA infection by inducing M1 macrophages

**DOI:** 10.1038/s41598-017-17001-0

**Published:** 2017-12-04

**Authors:** Yang Zhang, Xiang-Xiang Li, Yuan Ma, Jie Xu, Li-Na Zhao, Xue-Feng Qian, Xian-Feng Zhang, Jin-Fang Shi, Qing-Zhen Han

**Affiliations:** Center for Clinical Labrotary, the First Affiliated Hospital of Soochow University, 188# Shizi Road, Suzhou, 215006 Jiangsu China

## Abstract

*Corynebacterium pyruviciproducens* (*C. pyruviciproducens*, CP), as a newly discovered immunomodulator, has been confirmed to have a stronger immunoregulation than *Propionibacterium acnes* (*P. acnes*) of the traditional immune adjuvant, by previous experiments with model antigen ovalbumin and sheep red blood cells. Here, it was designed to assess its ability to resist methicillin-resistant *Staphylococcus aureus* (MRSA), since MRSA as a vital gram positive pathogen is characterized by high morbidity and mortality. In this report, it was indicated that *C. pyruviciproducens* and its peptidoglycan (CP-PGN) could help to be against bloodstream infection of MRSA with raised survival rate, decreased bacteria load and alleviated systemic inflammation, and these effects of CP-PGN were more pronounced. However, the whole CP was inclined to prevent localized abdominal infection of MRSA from progressing to a systemic infection. And they showed the potential as a therapeutic drug alone or combined with vancomycin. The diversity of capacity of activating macrophages induced by CP and CP-PGN may result in distinct resistance to MRSA in different infection models. Furthermore, both CP and CP-PGN induced M1 macrophages. In conclusion, CP and its PGN could act as promising immune agents to treat and prevent MRSA infection.

## Introduction

*C. pyruviciproducens* is a newly discovered bacterial immunoregulation without known pathogenic components. *C. pyruviciproducens* showed a stronger immunomodulatory effect than *P. acnes* of a well-known traditional immune adjuvant, confirmed by experiments with different types of pattern antigens, such as ovalbumin and sheep red blood cell^1–3^. This report is to investigate whether *C. pyruviciproducens* has a strong ability to resist infection caused by clinical pathogenic microorganisms, like *Staphylococcus aureus* (*S. aureus*).

Methicillin-resistant *S. aureus* (MRSA) can cause severe bacteremia and sepsis with high morbidity and mortality in hospitals and in the community^4^. Indeed, the death rate from MRSA sepsis remains greater than 20% due to uncontrolled inflammation and drug resistance^5^. In the treatment of MRSA infection, vancomycin remains the principal agent of choice. However, vancomycin use can be limited by its various shortcomings, including strong side effects, poor tissue penetration, slow bacterial killing^6^. In addition, repeated vancomycin treatment can increase the risk of developing resistance to critical antibiotic. These challenges necessiate the research for novel therapeutic approaches for MRSA infection. One possible strategy to modulate the host systemic anti-bacterial response and improve treatment outcomes is to combine antibiotic therapy with suitable immunomodulator^7–10^, since the development of new antibiotics is making a slow progress.

As we all know, macrophages occupy a unique niche in the immune system. They are effector cells that contribute to fight infection and inflammation^11^. On the one hand, macrophages exhibit a broad range of immunoregulatory activities and arise in response to different stimuli, especially bacteria or other particulate antigens^12^. On the other hand, macrophages are thought to be the important target cells of some immunomodulatory drug. They are the reasons why used the macrophages for the current experiments.

In the present experiments, firstly, we established the mouse model of bloodstream infection and local infection caused by MRSA to explore the potential preventive effect of *C. pyruviciproducens*. And two treatment models with CP alone or combined with vancomycin were also designed to determine its therapeutic effect for MRSA infection. Treating with or without the heat-inactivated *C. pyruviciproducens*, we mainly observed the survival situation, bacterial load, organs damage and inflammation of mice to evaluate the anti-MRSA-induced sepsis efficacy of *C. pyruviciproducens*. Secondly, we focused on the role of *C. pyruviciproducens* in regulating the function and differentiation of macrophages. What’s more, to reduce the side effects and improve its ability of anti-infection of heat-inactivated *C. pyruviciproducens*, here we extracted the peptidoglycan of *C. pyruviciproducens* (CP-PGN) and explored its ability to help resist MRSA infection and the possible mechanisms synchronously. In conclusion, CP and CP-PGN could protect mice against MRSA as potential preventive and therapeutic drugs but with different advantages in various infection models, that may be due to diversity of capacity of activating macrophages induced by CP and CP-PGN, although both of them induced M1 macrophages.

## Results

### Extraction and qualitative analysis of the CP-PGN

**Figure 1:**
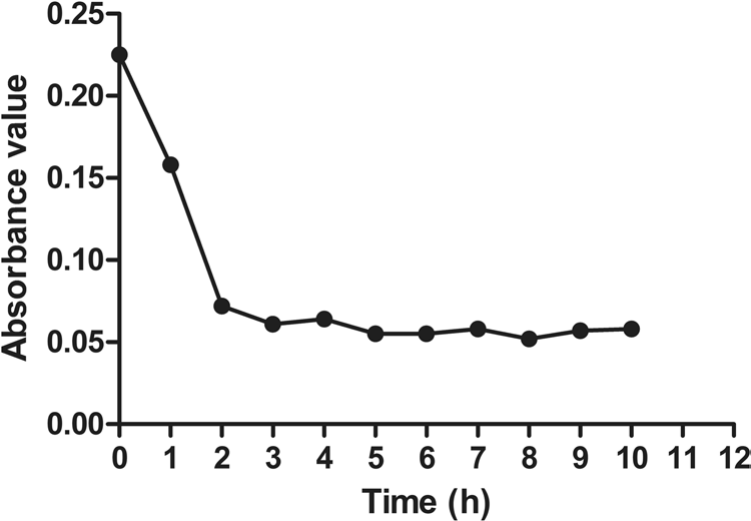
Qualitative analysis of the PGN of *C. pyruviciproducens* (CP-PGN). A_450nm_ of CP-PGN was determined in the degradation model of CP-PGN when exposed to lysozyme. Each graph represents the average of more than three replications.

Extraction of CP-PGN was processed according to TCA method. Peptidoglycan is chiefly composed of chains of *N*-acetylglucosamine (GlcNac) and *N*-acetylmuramic acid (MurNac) attached to stem peptides. In order to verify whether the product is the target, lysozyme assay was used, because the β-1,4-linked GlcNac and MurNac structure in CP-PGN can be exclusively hydrolyzed by lysozyme^13^. The absorbance values declined during the first 2 h, whereas no obvious dropping was observed by the following time (Fig. 1). The results suggested that CP-PGN possessed the characteristics of peptidoglycan.

### In the mouse model of bloodstream infection, *C. pyruviciproducens* and CP-PGN enhanced the ability to be against MRSA by promoting immune response

**Figure 2:**
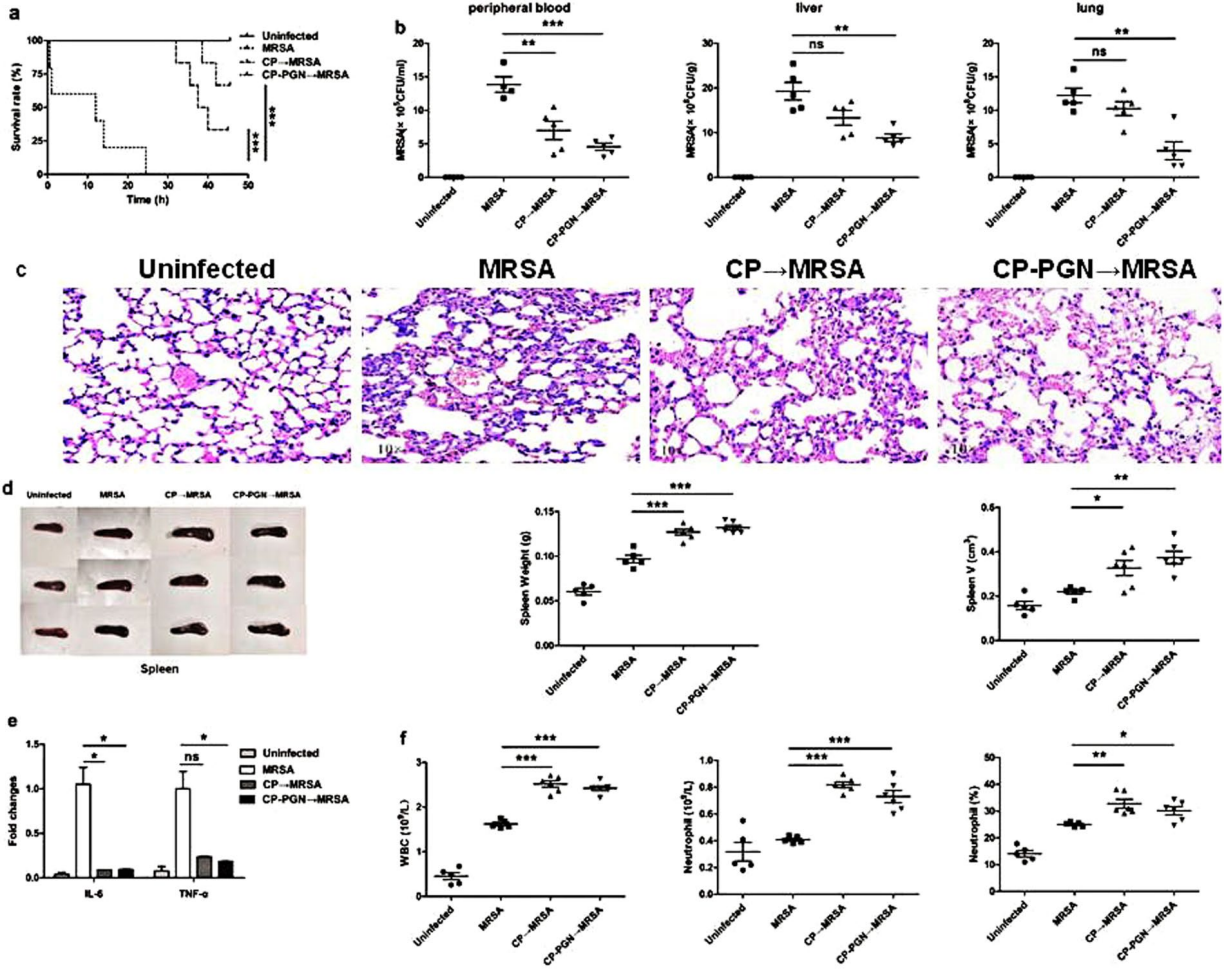
CP and CP-PGN extended the survival time of mice and enhanced the ability of anti-infection. In bloodstream infection model of MRSA, the survival rate (**a**) was increased compared with MRSA group. The bacterial CFU in peripheral blood, liver and lung (**b**) was decreased in the CP and CP-PGN group. Lungs (**c**) of all the groups were obtained analyzed by pathological sections and staining. CP and CP-PGN showed a more obvious effect of alleviated inflammation induced by MRSA. The weight and volume of spleen (**d**), inflammatory cytokines (**e**), leukocytes, neutrophils and neutrophil percentages (**f**) of blood of CP and CP-PGN pre-stimulated mice were determined compared with control group. Kaplan–Meier suvival curves were used to estimate survival rate. These data are representative of three independent experiments with similar results (n = 5–8 mice/group). The significant differences compared with the control group were analyzed by Student’s t test, ^*^ *P* < 0.05, ^**^ *P* < 0.01, ^***^ *P* < 0.001 vs. control. CP = *C. pyruviciproducens*; CP-PGN = Peptidoglycan of *C. pyruviciproducens*.

To investigate the ability of anti-infection of *C. pyruviciproducens* and CP-PGN, a mouse model of anti-MRSA was studied. It was found that in the MRSA group, all the mice died in 25 h. While in the CP and CP-PGN group, the first mouse died in 32 h and 38 h respectively. Furthermore, we found that, in the CP and CP-PGN groups, there still had some mice survived, especially for the CP-PGN group (Fig. 2a). And markedly decreased CFU of MRSA was determined in peripheral blood, liver and lung (Fig. 2b).

As well known, the final death induced by MRSA sepsis is a result of the uncontrolled inflammatory destruction of vital organs. Remarkably, in the model of bloodstream infection, CP and CP-PGN played a great role of alleviating the inflammation of some vital organs induced by MRSA, like the lungs (Fig. 2c). And PGN of *C. pyruviciproducens* showed a more pronounced effect of reducing inflammation induced by MRSA than the whole CP.

In addition, the spleen is a vital immune organ and has important effects on the prognosis of sepsis specially^14^. So in the model of bloodstream infection caused by MRSA, the weight and volume of spleen from *C. pyruviciproducens* or CP-PGN stimulated mice were obviously increased compared with control group (Fig. 2d). And more innate immune cells were attracted to peripheral blood to phagocytize the bacteria with the help of CP and CP-PGN (Fig. 2e).

All these results suggested that CP and CP-PGN played a great role in resisting MRSA bloodstream infection by attracting innate immune cell and reducing the destructive inflammation of vital organs, especially for PGN of *C. pyruviciproducens*.

### In the model of localized abdominal infection, *C. pyruviciproducens* and CP-PGN could prevent local MRSA infection from progressing to a systemic infection

**Figure 3:**
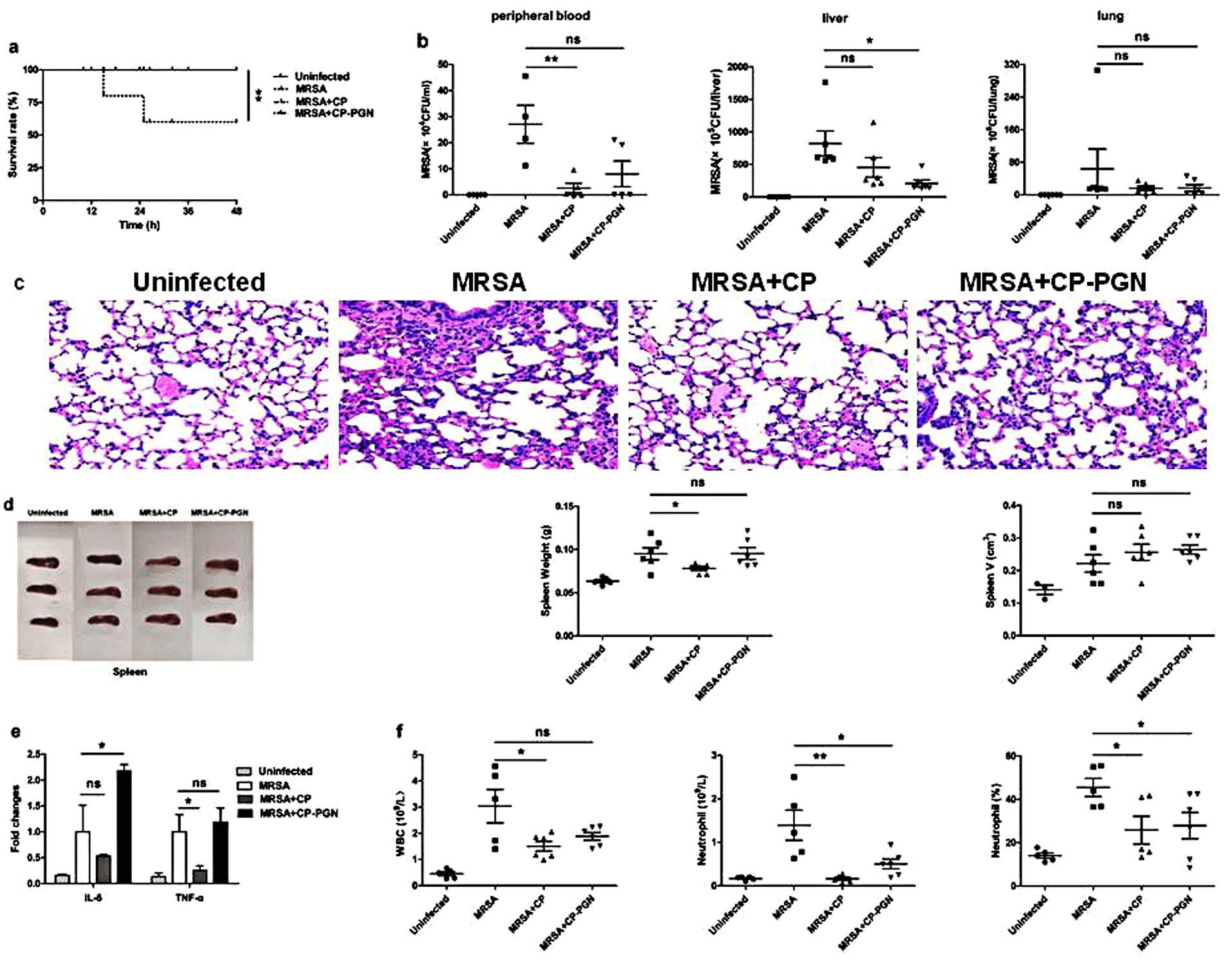
CP and CP-PGN prevented local MRSA infection from progressing to a systemic infection. In a local abdominal infection model, the survival rate (**a**) was improved, bacterial load (**b**) was reduced and inflammatory damage of lung (**c**) was alleviated compared with MRSA group. The weight and volume of spleen (**d**) were changed not obviously. The inflammatory factors (**e**) and leukocytes (**f**) in peripheral blood were depressed more obviously in CP group than MRSA group. Kaplan–Meier suvival curves were used to estimate survival rate. These data are representative of three independent experiments with similar results (n = 5–8 mice/group). The significant differences compared with the control group were analyzed by Student’s t test, ^*^ *P* < 0.05, ^**^ *P* < 0.01, ^***^ *P* < 0.001 vs. control. CP = *C. pyruviciproducens*; CP-PGN = Peptidoglycan of *C. pyruviciproducens*.

In the localized abdominal MRSA infection, stimulation of CP or CP-PGN could prevent local infection from progressing to systemic infection, with improved survival rate (Fig. 3a), reduced bacterial load (Fig. 3b) and alleviated inflammatory damage of lung (Fig. 3c). Maybe because CP and CP-PGN were injected by local administration, the change of spleen is not obvious (Fig. 3d). In addition, inflammatory factors (Fig. 3e) and white blood count (Fig. 3f) in peripheral blood were depressed more obviously in CP group than MRSA group. All these results indicated that although CP and CP-PGN improved the same level of survival of mice with abdominal infection, the whole cell of heated CP seemed to be more effective in limiting local infection of MRSA than its PGN.

### In the therapeutic model of mild MRSA infection, *C. pyruviciproducens* and CP-PGN showed the same treatment effect as vancomycin

**Figure 4:**
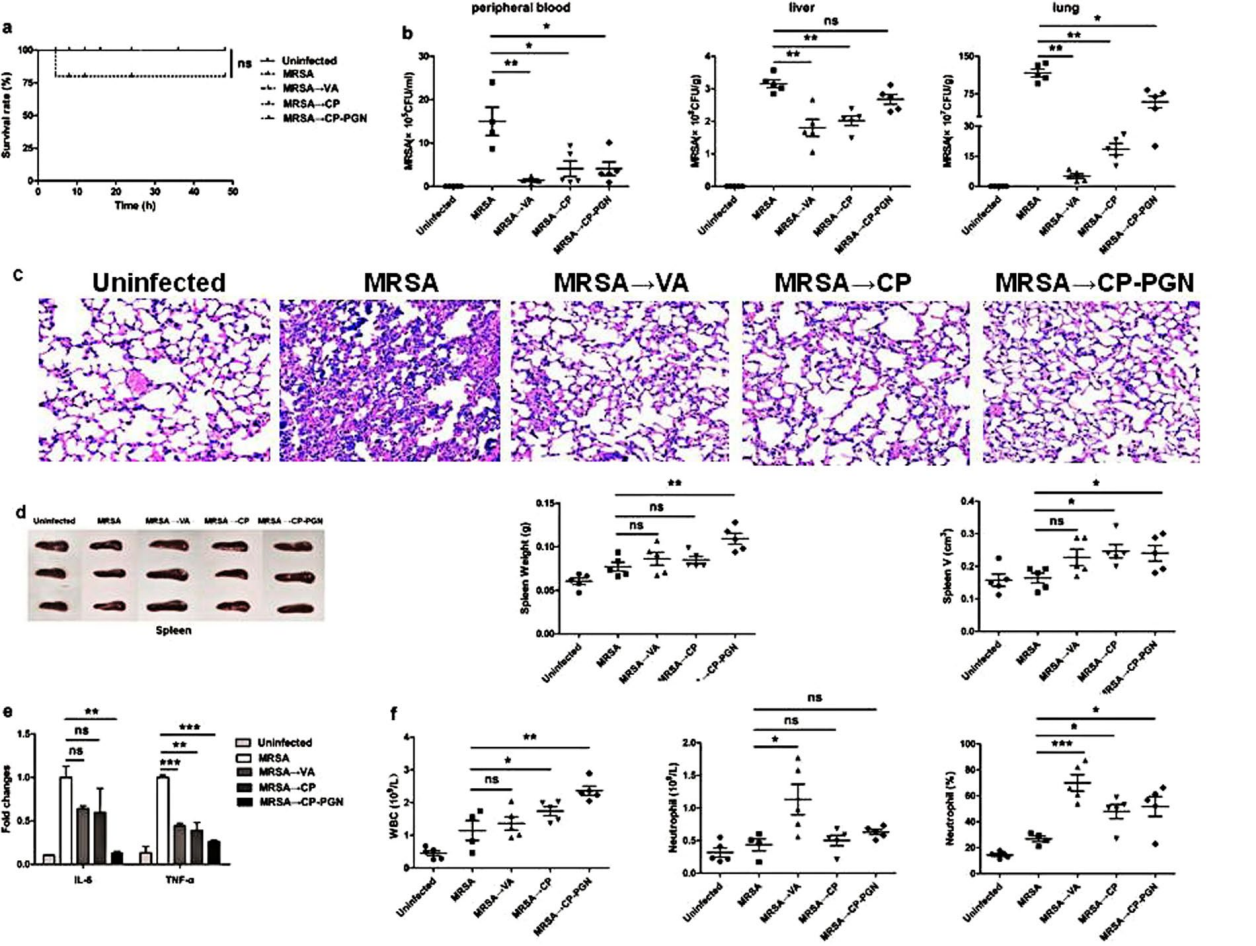
*C. pyruviciproducens* and CP-PGN showed the same treatment effect as vancomycin. In the therapeutic model of MRSA infection, the survival rate (**a**) in CP and CP-PGN group were raised. The bacterial load (**b**) was reduced more obviously with vancomycin. The changes of lung (**c**) and spleen (**d**) was no differences between CP or CP-PGN and vancomycin. CP-PGN had stronger ability to depress inflammatory cytokine level (**e**). The total number of white blood cells in blood arised more in all the treatment group than MRSA infection Untreated, but the increase was more due to neutrophils in vancomycin group (**f**). Kaplan–Meier suvival curves were used to estimate survival rate. These data are representative of two independent experiments with similar results (n = 5–8 mice/group). The significant differences compared with the control group were analyzed by Student’s t test, ^*^ *P* < 0.05, ^**^ *P* < 0.01, ^***^ *P* < 0.001 vs. control. VA = Vancomycin; CP = *C. pyruviciproducens*; CP-PGN = Peptidoglycan of *C. pyruviciproducens*.

The administration of vancomycin, CP or CP-PGN alone after MRSA infection could raise survival rate with no mice died compared with the untreated MRSA infection group, although there was no statistical difference (Fig. 4a). On reducing bacterial load, vancomycin has the strongest effect (Fig. 4b). However, CP-PGN seemed to be more powerful on the depressed inflammatory cytokine level after MRSA infection than vancomycin (Fig. 4e). And more white blood cells were attracted into peripheral blood by CP or CP-PGN, but it seemed to be just more neutrophils attracted by vancomycin (Fig. 4f). Furthermore, there was no significant differences on data of inflammatory destruction of lung (Fig. 4c) and changes of spleen (Fig. 4d) between CP or CP-PGN and vancomycin, although such significances were obvious compared with the untreated MRSA infection group.

### In the therapeutic model combined with vancomycin, *C. pyruviciproducens* and CP-PGN improved the survival rate after MRSA infection

**Figure 5:**
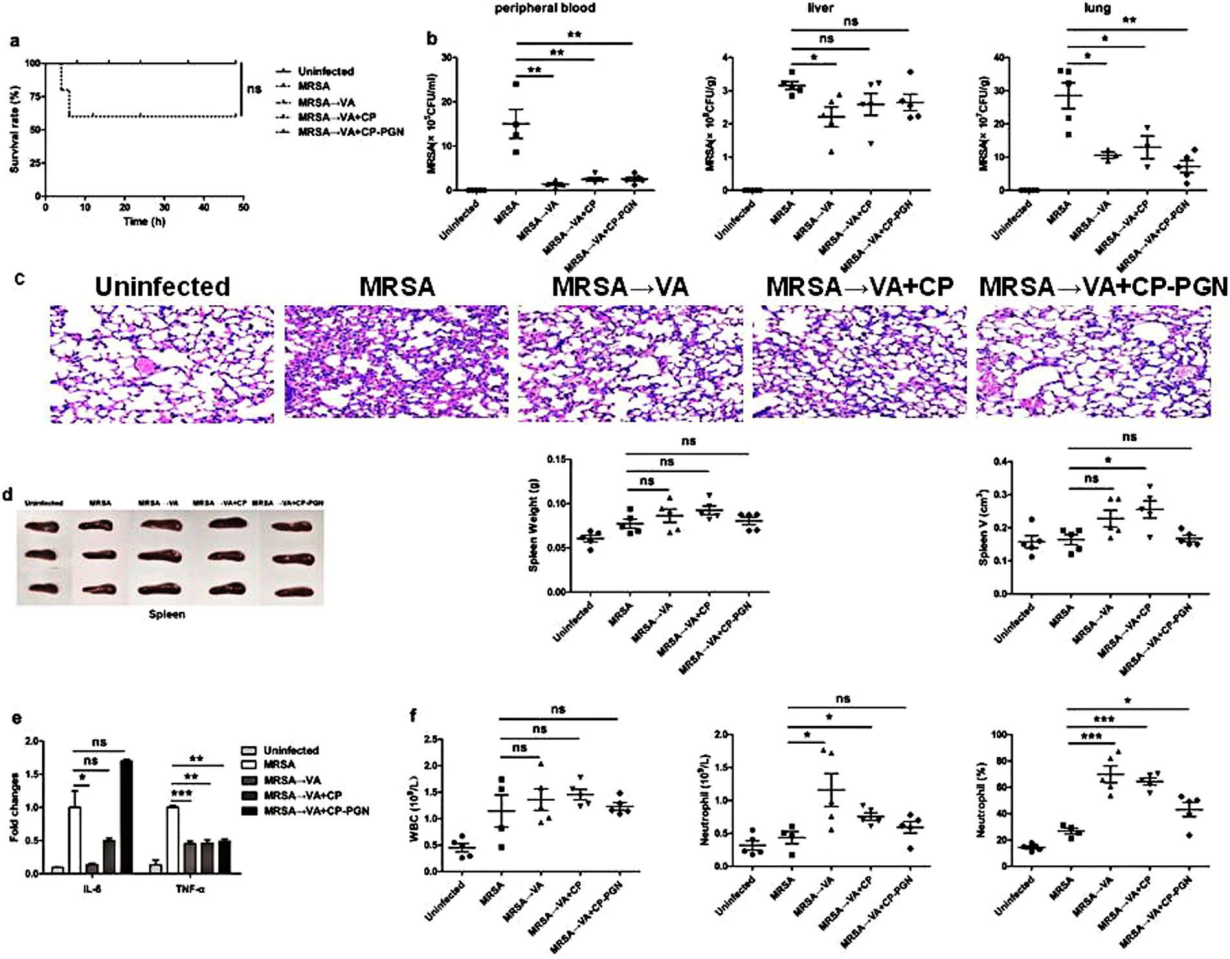
CP or CP-PGN combined with vancomycin could improve the survival rate and condition. In the CP and CP-PGN combined with vancomycin group, survival state was improved (**a**), bacterial load was reduced (**b**) and the degree of lung damage were alleviated (**c**) compared with the untreated MRSA infection group. There was no obvious significance on the changes of spleen (**d**). The level of IL-6 was not inhibited obviously by the treatment of vancomycin and CP-PGN (**e**). More neutrophils were attracted into blood by vancomycin alone than a combination with CP or CP-PGN (**f**). These data are representative of two independent experiments with similar results (n = 5–8 mice/group). The significant differences compared with the control group were analyzed by Student’s t test, ^*^ *P* < 0.05, ^**^ *P* < 0.01, ^***^ *P* < 0.001 vs. control. VA = Vancomycin; CP = *C. pyruviciproducens*; CP-PGN = PGN of *C. pyruviciproducens*.

The same survival state was improved (Fig. 5a), the similar level of bacterial load was reduced (Fig. 5b) and the same degree of organ damage were alleviated (Fig. 5c) compared with the untreated MRSA infection group, no matter using vancomycin or combined with CP or CP-PGN. And no significant differences were found on changes of spleen (Fig. 5d) and inflammatory factors (Fig. 5e) between vancomycin and a combination with CP or CP-PGN. However, a combination with CP or CP-PGN could cut down the number and proportion of neutrophils compared with vancomycin (Fig. 5f).

### CP and CP-PGN could effectively activate macrophages

**Figure 6:**
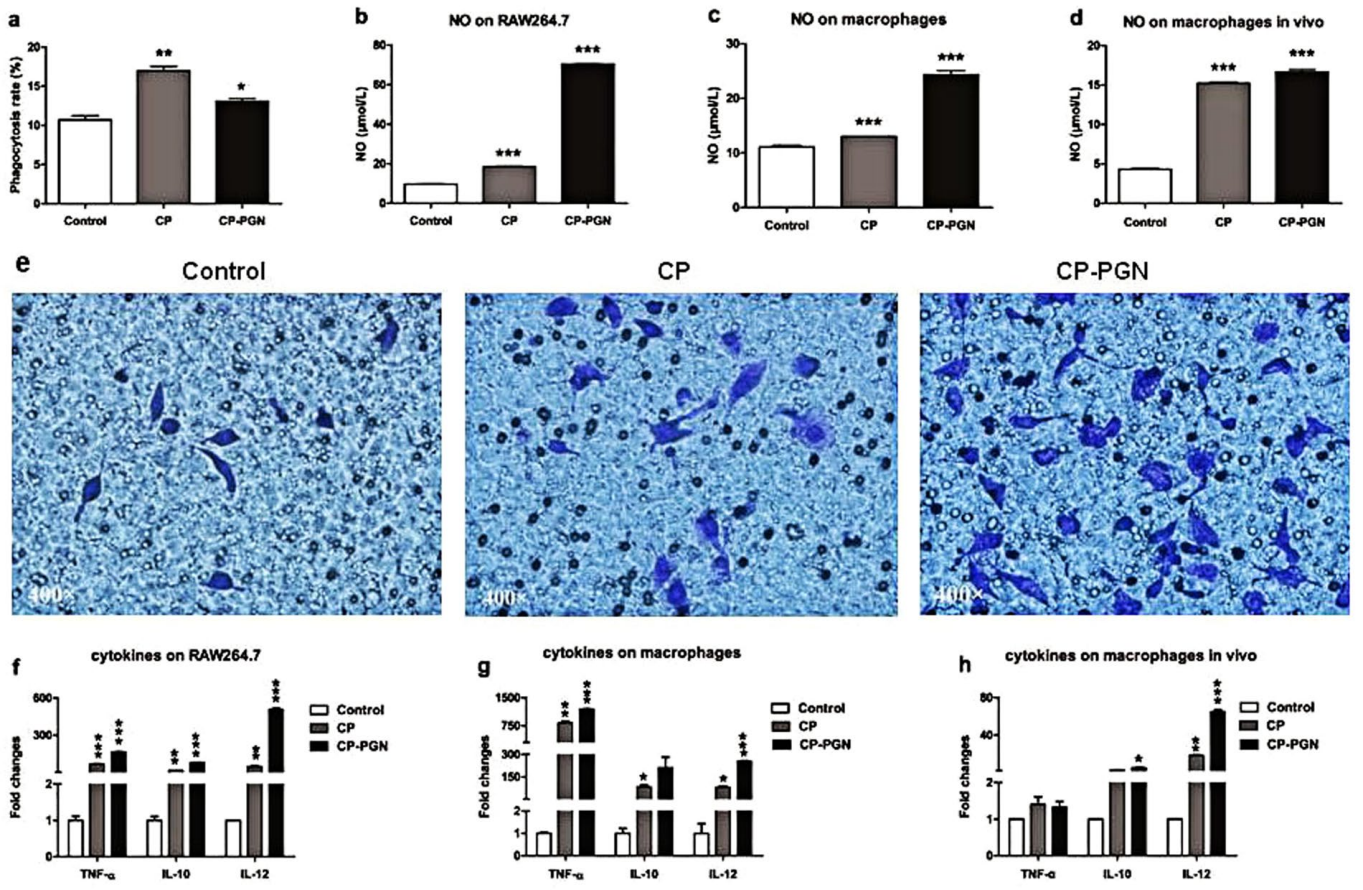
CP and CP-PGN could activate macrophages *ex vivo* and *in vivo*. With CP and CP-PGN pretreated RAW264.7 cells co-stimulated with MRSA, they showed higher phagocytosis rate (**a**) than the control. The production of NO both in RAW264.7 cells (**b**), peritoneal macrophages (**c**) and macrophages *in vivo* (**d**) was increased under the treatment of CP or CP-PGN. More RAW264.7 cells made migrating (**e**) induced by CP or CP-PGN, especially for CP-PGN, compared with control group. The expression of cytokines on RAW264.7 (**f**), macrophages (**g**) and macrophages *in vivo* (**h**) was higher in the group of CP or CP-PGN. These data are representative of at least three experiments. The significant differences compared with the control group were analyzed by Student’s t test, ^*^ *P* < 0.05, ^**^ *P* < 0.01, ^***^ *P* < 0.001 vs. control. CP = *C. pyruviciproducens*; CP-PGN = PGN of *C. pyruviciproducens*.

To explore the activation of macrophages by stimulation of CP and CP-PGN, a series of functional tests were carried out including phagocytosis and chemotaxis test, and detection of killing factors and cytokines *ex vivo* and *in vivo*. The whole *C. pyruviciproducens* performed better to promote phagocytosis of MRSA by macrophages than its PGN *ex vivo* (Fig. 6a). However, CP-PGN indicated greater power to induce chemotaxis (Fig. 6e) and expression of NO (Fig. 6b–d) and cytokines (Fig. 6f–h) on macrophages *ex vivo* or *in vivo* than the whole CP, although the later also acted well.

### CP and CP-PGN skewed macrophages to a M1-like type

**Figure 7:**
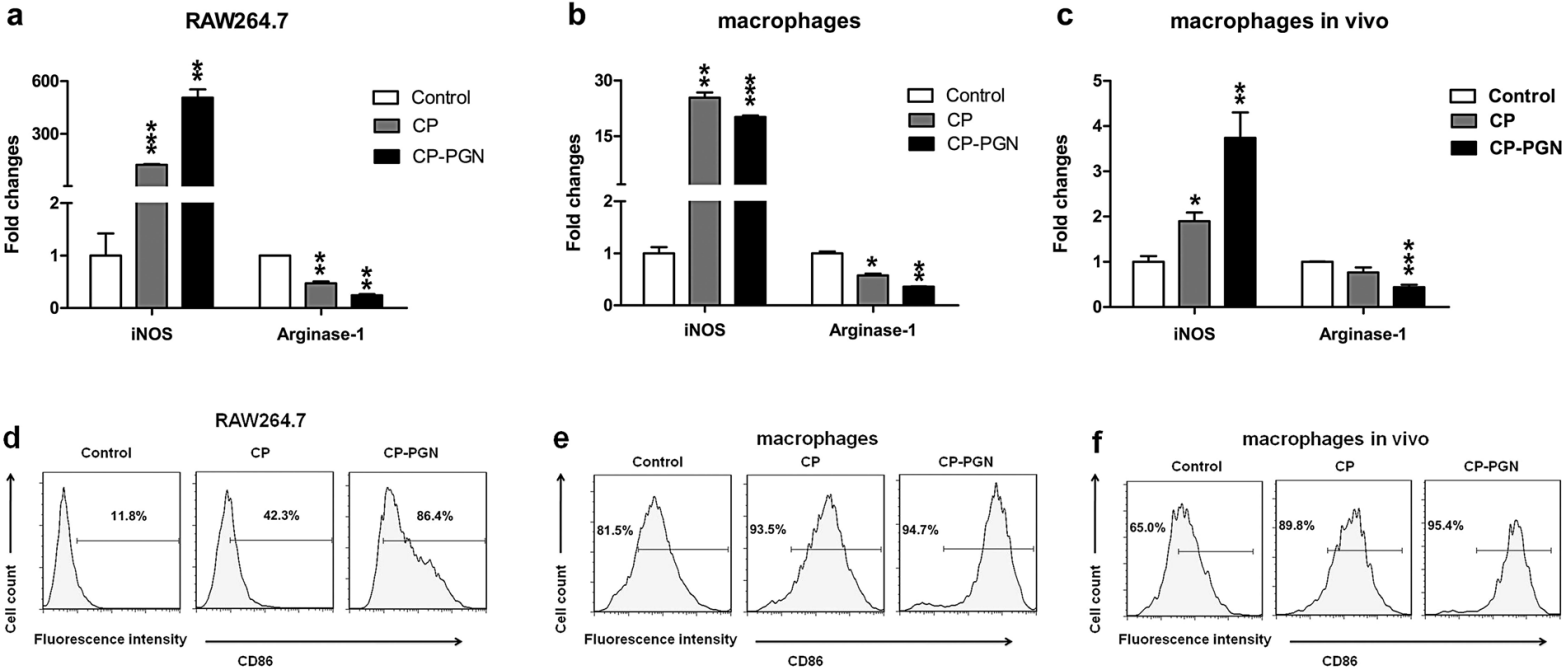
CP and CP-PGN skewed macrophages to a M1-like type. RAW264.7 cells (**a,d**) and peritoneal macrophages (**b,e**) were co-cultured with CP or CP-PGN *ex vivo* to detect the iNOS and arginase-1 by qRT-PCR and CD86 by Flow cytometric analysis. Macrophages pretreated with CP or CP-PGN for 24 h *in vivo* (**c,f**) were collected from the abdominal cavity of mice to analyzed iNOS, arginase-1 and CD86. Data shown are representative of three independent experiments. The significant differences compared with the control group were analyzed by Student’s t test, ^*^ *P* < 0.05, ^**^ *P* < 0.01, ^***^ *P* < 0.001 vs. control. CP = *C. pyruviciproducens*; CP-PGN = PGN of *C. pyruviciproducens*.

The polarization of macrophages directly determines their function, since M1 macrophages mainly play a role in resisting bacteria and M2 macrophages help with repairing tissue under the uninfected circumstances. Therefore, the potential for regulating differentiation of macrophages was investigated. Higher levels of iNOS and CD86, as main signs of M1 macrophages were detected to be raised after stimulation of CP and CP-PGN in RAW264.7 cells (Fig. 7a,d) and primary macrophage and peritoneal macrophages (Fig. 7b,e) *ex vivo*. Data of experiments *in vivo* presented similar results (Fig. 7c,f). As a whole, CP and CP-PGN were more inclined to induce M1 macrophages.

### Silencing of TLR2 down-regulated CP/CP-PGN-induced activation and polarization in RAW264.7 cells

**Figure 8:**
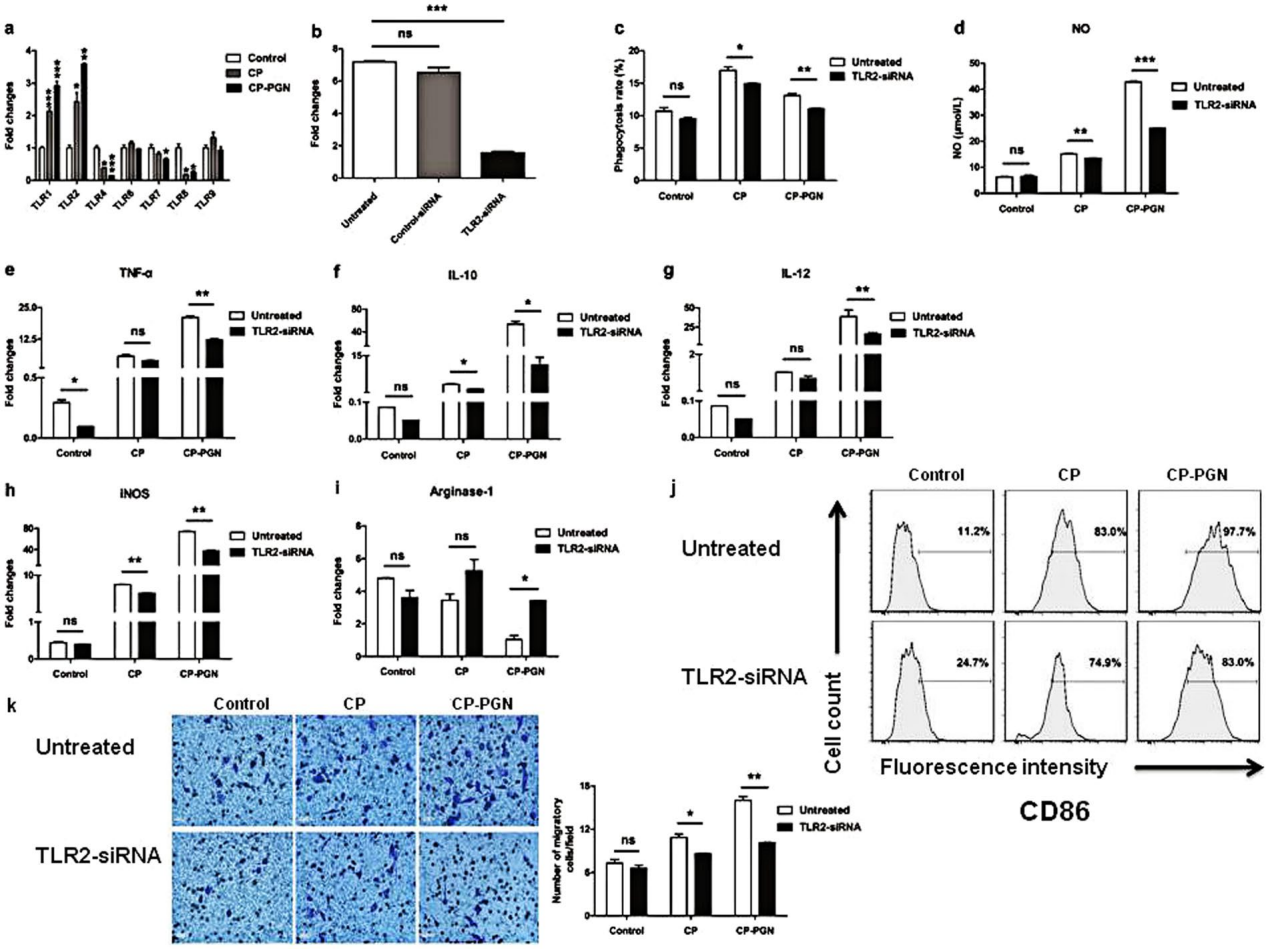
Silencing of TLR2 down-regulated CP/CP-PGN-induced activation and differentiation of RAW264.7 cells. The expression levels of TLRs were analyzed by qRT-PCR to select out which TLR changed most obviously (**a**). RAW264.7 cells transfected 24 h with TLR2 siRNA/control siRNA, then the mRNA of TLR2 were assayed by qRT-PCR (**b**). CP and CP-PGN pretreated RAW264.7 or RAW264.7 transfected with TLR2 siRNA co-stimulated with MRSA to analysis the phagocytosis rate (**c**). RAW264.7 or RAW264.7 transfected with siTLR2 were co-cultured with CP or CP-PGN for 24 h to detect the production of NO (**d**), for 3 h to detect cytokines (**e,f,g**), iNOS (**h**) and arginase-1 (**i**) by qRT-PCR. CD86 were determined by flow cytometric analysis (**j**) and cell migration assay was shown (**k**). Each graph represents the average of three replications. The significant differences compared with the untreated group were analyzed by Student’s t test, ^*^ *P* < 0.05, ^**^ *P* < 0.01, ^***^ *P* < 0.001 vs. untreated. CP = *C. pyruviciproducens*; CP-PGN = PGN of *C. pyruviciproducens*.

The expression level of TLRs about RAW264.7 stimulation of CP and CP-PGN (Fig. 8a) showed that TLR2 changed most significantly. To further determine whether TLR2 mediated CP/CP-PGN-induced activation and polarization in RAW264.7, the cells were stimulated with CP/CP-PGN after transfection with TLR2 siRNA. The data showed that, the expression level of TLR2 decreased notably in RAW264.7 cells transfected with TLR2 siRNA compared with untreated cells (Fig. 8b). A series of functional tests as described above were carried out subsequently to investigate the function of TLR2. The results showed that, the phagocytosis (Fig. 8c) and chemotaxis (Fig. 8k), the production of NO (Fig. 8d), cytokines (Fig. 8e–g), iNOS (Fig. 8h), arginase-1 (Fig. 8i) and CD86 (Fig. 8j) were all reduced obviously after stimulation of CP and CP-PGN in TLR2 siRNA- transfected cells compared with untreated cells, especially for stimulation by CP-PGN. On the contrary, the expression of arginase-1 (Fig. 8i) was increased by CP/CP-PGN on the TLR2 siRNA-transfected cells, due to its effect of down-regulalted expression suppressed by the silencing of TLR2.

## Discussion

With the increasing chronic and immunodeficiency diseases, the incidence of *S. aureus* infections has been increased dramatically around the world. On the one hand, multidrug resistance of MRSA exacerbated the global antibiotic crisis^15^. On the other hand, MRSA, as a major cause of bloodstream infections in hospitals, is a leading cause of life-threatening nosocomial infections, being associated with high mortality rate, prolonged length of hospital stay and a substantial economical burden on health care systems^16^. Therefore, it’s urgent to study new therapeutic agents and develop effective preventive therapies. In this report, it was demonstrated that pretreated with *C. pyruviciproducens* or CP-PGN, especially the later, could significantly improve the ability to resist the bloodstream infection of MRSA, with raised overall survival rate, extended the survival time, less bacterial load in the body, reduced inflammation cytokines and alleviated inflammatory damage to vital organs. All of that benefitted not only from recruiting many neutrophils to the peripheral blood to participate in the phagocytosis and clearance of bacteria, but from enhanced immunity with the help of *C. pyruviciproducens* or CP-PGN^17^, since rapid removal of bacteria and reduced systemic destructive inflammation were essential to depress the pathogenicity of sepsis^18^. Therefore, to improve the patient’s resistance to pathogens, CP-PGN could be used as a potential preventive drug against MRSA before surgery or during hospitalization, especially for those immunocompromised people. For example, people with chronic disease like diabetic and Hypertension, radio therapy and chemotherapy patients for the cure of cancers and children or the aged with low immunity.

Surprisingly, *C. pyruviciproducens* and CP-PGN significantly blocked spreading of MRSA from the abdominal cavity to the whole body in the local abdominal infection model, with markedly improved survival rates compare with the untreated mice. But data of CFU in the peripheral blood, inflammation of the lungs, and the inflammatory factors in blood, indicated that the whole CP acted more strongly. Why does PGN play a stronger role in preventing bloodstream infections and the whole *C. pyruviciproducens* act more powerfully in limiting the local infection? Maybe, it’s partly because the whole *C. pyruviciproducens* indicated stronger ability to promoting phagocytosis, but peptidoglycan of *C. pyruviciproducens* presented stronger chemotaxis and activation of macrophages by the following experiments on macrophages *ex vivo* and *in vivo*.

To test the therapeutic effect of CP and CP-PGN for MRSA infection, two animal models were established, one was to treat infection using CP or CP-PGN alone after inntravenous injection of MRSA, and the other using CP or CP-PGN combined with vancomycin. CP or CP-PGN showed the same power as vancomycin in improving survival rates with only some mice died in MRSA group without any treatment and none of other group dead. However, the bacterial load decreased most markedly under the administration of vancomycin among all the groups in both therapeutic models. After all, vancomycin is an antibiotic that directly kills MRSA. Instead, the advantage of CP and CP-PGN is that they act as a role of immune adjuvant to indirectly help remove the bacteria by mobilizing the body’s own immune system, which could be verified by white blood count with more leukocytes and a smaller percentage of neutrophils in CP or CP-PGN group than vancomycin group, in another word, more other effective immune cells like lymphocytes and monocytes were attracted to the blood by CP or CP-PGN. Anyhow, CP or CP-PGN may be a potential drug alone for cure of mild MRSA infection, and in severe infection, the dosage of antibiotics may be reduced by using a combination of CP or CP-PGN but antibiotics is irreplaceable.

It is common knowledge that macrophages play a key role in innate immunity and initiate adaptive immune response as antigen presenting cells^11,19,20^. In anti-infection immunity, macrophages can directly eliminate pathogenic microorganisms by phagocytosis and producing many effectors like NO. At the same time, equally important is the ability to secrete cytokines and chemokines to recruit other immune cells to trigger an effective pathogen-eliminating response^21^. Our study provided further evidence that both *C. pyruviciproducens* and CP-PGN could activate macrophages *ex vivo* and *in vivo*. However, heated-inactivated *C. pyruviciproducens* were mainly to enhance the phagocytosis of macrophages, and CP-PGN played a more important role in promote macrophages to release lots of nitric oxide (NO), an important inflammatory mediator^22^. The main reason for the difference may be related to the different ways they interact with macrophages. On the one hand, *C. pyruviciproducens*, as a particulate matter, depend more on direct swallowing. On the other hand, the molecular CP-PGN as a kind of PAMP is mainly recognized by the corresponding PRRs, such as TLR2 and nucleotide-binding oligomerization domain (Nod) proteins^23,24^. The two ways to contact with macrophages are equally important at resisting microbial infections^25^. As shown by data *in vivo*, both the whole *C. pyruviciproducens* and its PGN acted well during the period of MRSA infection, but with their advantages in local infection and systemic infection respectively.

In response to environmental signals, macrophages undergo polarization towards a proinflammatory (M1) or anti-inflammatory (M2) phenotype. M1 macrophages promote and amplify Th1 type responses. The typical characteristics of M1 macrophages include strong antigen presentation, producing high levels of proinflammatory cytokines, reactive nitrogen and oxygen intermediates^26^. They are endued with the capacity to and implicated in the host defense against intracellular pathogens and possess antitumor activity^27^. In contrast, M2 macrophages are the “resting” phenotype with promotion of Th2 response can induce immune tolerance and tumor progression. They are characterized by their high phagocytic capacity and releasing anti-inflammatory cytokine that promote the proliferation of contiguous cells and tissue repair^26,28,29^.

M1 macrophages play an important role in the process of anti-infection. iNOS, TNF-α, IL-12 and CD86 are specific for M1 subtypes, while arginase-1, Dectin-1 and mannose receptor are specific for M2 subtypes. In fact, the main markers used frequently in experiments were iNOS and arginase-1^30,31^. The experiments *ex vivo* and *in vivo* showed that both *C. pyruviciproducens* and CP-PGN could significantly up-regulate iNOS and down-regulate arginase-1 expression in RAW264.7 cells and peritoneal macrophages, which skewed macrophages toward M1 phenotype. In addition, CD86, another M1 macrophages cell surface marker, also showed a high expression level on the surface of macrophages stimulated with *C. pyruviciproducens* or CP-PGN. Collectively, these results indicated that *C. pyruviciproducens* and CP-PGN promoted differentiation of macrophages into the classic M1 phenotypes, so as to enhance the ability of anti-infection.

It is generally accepted that the peptidoglycan (PGN) of bacteria is the ligand of TLR2. In our study, RNAi technology was used to silence TLR2 gene expression in RAW264.7 cells. Our data suggested that silencing of TLR2 could affect the activation and polarization of RAW264.7 cells induced by CP and CP-PGN, while CP-PGN performed more obviously. In the point of view, it was because that, there are many kinds of ligands to react with TLRs on heat-inactivated CP as the whole cell, not just only with TLR2. However, CP-PGN as a pure composition of CP is just the ligand of TLR2. Therefore, it was verified that CP-PGN functioned as immunoregulation mainly by the combination with TLR2 appeared on the cell surface. Why could CP-PGN play the role of anti-infection through the TLR2, instead of peptidoglycan of other bacteria? To make a better and professional explanation, many further studies were needed to carry out, such as to analysis the structure of CP-PGN and the profound downstream mechanism of CP-PGN combined with TLR2.

Antibiotics are becoming ineffective owing to rapid and widespread emergence of multidrug resistant bacteria. Under the global antibiotic crisis, there is an urgent demand for new agents and novel therapeutic strategies to address this challenge. Immunomodulator can be a promising adjuvant tool for the prevention and treatment of infection to mitigate the issue of antibiotic resistance, especially for patients with chronic diseases and immunocompromised patients^32,33^. It was confirmed in our experiments that *C. pyruviciproducens* and CP-PGN significantly improved the ability of anti-MRSA infection. In order to achieve the better effect, it was suggested that *C. pyruviciproducens* was good for the local infection, in contrast, CP-PGN could be chosen for systemic blood infection to reduce the systemic injury. In mild infection, they may be used instead of antibiotics, but in severe infection a combination with suitable antibiotics would be recommended. Of course, further large-scale investigations should be performed to validate our findings before applying to the clinical trial. We expect that novel immune modulator would be able to reduce the burden of infective diseases worldwide.

## Methods

### Bacterial strains

*C. pyruviciproducens* (CCUG 57046, ATCC BAA-1742 obtained from Olive View Medicine Center, University of California, Los Angeles, CA, USA) was cultured for two days, then collected to be inactivated by heat as described before^2^.

### Animals

Female BALB/C mice (age 6 to 8 weeks) were purchased from Yangzhou University and acclimated for at least 1 week before use. All the measures taken for the mice were in accordance with approved guidelines (Guideline for the Care and Use of Laboratory Animals) established by the Chinese Council on Animals Care. In addition, all the animal experimental protocols were approved by the Ethics Committee of Soochow University (Suzhou, China).

### Extraction of peptideglyean

The extraction of CP-PGN is based on the TCA method^13^. The harvested bacterial sludge was washed in 0.9% saline solution until it turned milk white. The clean sludge was dissolved in 10% TCA (w/v = 1:10), incubated in a boiling bath for 20 min, then centrifuged at 4,000 rpm for 10 min. The sediment were washed with sterile water for 3 times and centrifuged at 4,000 rpm for 10 min. Collecting the sediment and treated with 8% SDS (Beyotime Biotechnology, Jiangsu, China), incubated in a boiling bath for 30 min, then centrifuged at 4,000 rpm for 30 min. After centrifugation, the sediment was washed in sterile water for 3 times. Adding the 1 mg/ml trypsin (Amresco, USA) buffer to the above sediment at 37 °C in a shaking bath (240 rpm) for 3 hours, centrifuged at 3,000 rpm in 4 °C for 5 min, then collected the supernatant. The collecting supernatant was centrifuged at 12,000 rpm for 15 min. The sediment was added to aether and rested for 30 min, then centrifuged for 15 min to collect sediment. The harvested sediment were finally added to absolute alcohol and drying at 70 °C to get the hazel target product, then stored in an airtight container at −20 °C for further analysis.

### Analytical Methods of CP-PGN

For determination whether the hazel sediment was target product, CP-PGN was treated with lysozyme (200 μg/ml, dissolved in 0.1 mol/l PBS, PH6.2) (Amresco, USA) at 37 °C in a shaking bath (140 rpm). Lysozyme was treated identically for comparison. Collecting the reactant every hour, measured its UV absorbance at 450 nm in a Microplate Reader.

### RAW264.7 and peritoneal macrophages

The RAW264.7 cell line (kept by our laboratory) was cultured in RPMI1640 medium containing 10% heat-inactivated FBS at 37 °C, 5% CO_2_ atmosphere. Peritoneal macrophages were derived from BALB/c mice, referring to the previous reports^3^. All the experiments were divided into three groups: control group; *C. pyruviciproducens* group (CP group); peptideglyean of CP group (CP-PGN group).

### Mouse model of infection caused by MRSA

A mouse model of bloodstream infection was modified from previous studies as described below: female BALB/c mice (age 6 to 8 weeks) were randomly assigned to four groups: (1) uninfected group (sterile PBS); (2) MRSA group; (3) CP group (500 μg/mouse); (4) CP-PGN group (100 μg/mouse). Mice were injected with PBS, CP or CP-PGN in PBS intravenously (i.v.) one day before, then challenged by intravenous MRSA (2.5 × 10^10^/mouse). In another local abdominal infection model, after pre-treating with sterile PBS, CP or CP-PGN for 4 hours by intraperitoneal injection, mice were injected with MRSA (1 × 10^15^/mouse) intraperitoneally. In the therapeutic model of mild MRSA infection, mice were injected with MRSA (0.8 × 10^10^/mouse) intravenously, after 4 hours, mice in different groups were injected with vancomycin (30 mg/kg), CP (500 μg/mouse) or CP-PGN (100 μg/mouse) alone intravenously to treat infection. The uninfected mice were handled with PBS synchronously all through the whole process. Finally, in the therapeutic model combined with vancomycin, MRSA (1.8 × 10^10^/mouse) were injected intravenously. Vancomycin alone (30 mg/kg), vancomycin combined with CP (500 μg/mouse) or CP-PGN (100 μg/mouse) was applied intravenously to control infection. The uninfected mice were handled with the same way above.

After infection, mice were monitored daily and they were allowed free access to water and food throughout the experimental period. Mice were observed with survival state. Pathological changes of lung, CFU of MRSA in liver and lung and changes of spleen from the dead mice were detected. Those mice that survived for more than 48 hours were to be sacrificed to detect CFU of MRSA in blood, liver and lung, make pathological section of lung, measure the size of spleen, check the level of cytokines (IL-6 and TNF-α by qRT-PCR) in blood and count leucocytes in peripheral blood.

### Macrophage phagocytosis assay *ex vivo*

RAW264.7 cells were seeded in 12-well plates (1 × 10^6^/well) for 24 h before stimulation. The cells adhered to the bottom were kept by removal of suspension, then the new medium was added with heat-inactivated CP (200 μg/ml) or CP-PGN (50 μg/ml). After 3 h, the suspension was removed, added MRSA solution (3 × 10^5^/ml) for 3 h. Then cells were collected to make climbing tablets, and the phagocytosis rate was evaluated under the microscope through the gram staining.

### Griess assay *ex vivo*

Three groups were tested as described above. Nitric oxide production was determined by measuring its stable end product nitrite, using a Griess reagent (Beyotime Biotechnology, Jiangsu, China) according to manufacturer’s protocol. The RAW264.7 cells and peritoneal macrophages were pretreated with both CP (200 μg/ml) or CP-PGN (50 μg/ml) for 24 h, then 50 μl of supernatant was added to 96-well plate, followed by 50 μl Griess reagent I and 50 μl Griess reagent II at room temperature. Absorbance at 540 nm was measured by microplate reader. The concentration of NO_2_^−^ in the samples was calculated using a standard curve of sodium nitrite.

### Cell migration assay *ex vivo*

Cell migration was measured using a transfilter transwell assay using 24-well transwell chambers (Transwell, Corning Costar). 600 μl of medium with or without 200 μg/ml of heated-inactivated *C. pyruviciproducens* or 50 μg/ml of CP-PGN was placed in lower wells of chambers which were separated from 200 μl of cell suspension in the upper wells by polyvinylpyrrolidone-free polycarbonate filters (8.0um pore size, 6.5 mm diameter insert). Transwell chambers were incubated at 37 °C in 5% CO_2_ for 24 h. The upper surfaces of the filter membranes were washed three times with cold PBS to remove cells, which had settled on the membrane upper surface but not migrated. Cells trapped in the filter pores or adherent to the undersurface were fixed in cold methanol and stained with 0.1% Crystal Violet Staining Solution for counting. The total number of cells trapped in the pores was assessed for each well.

### qRT-PCR for detecting inflammatory cytokines

RAW264.7 cells were cultured in media containing 100/200/300/400/500 μg/ml heated-inactivated CP or 1/30/50/100/200 μg/ml CP-PGN for 3 hours. When the most appropriate concentration of CP or CP-PGN was selected, then treated with CP for 0.5/1/2/3/4/5/6/8/12 hours or CP-PGN for 0/1/3/8/12 hours to select the best stimulated time. RAW264.7 and peritoneal macrophages were stimulated with *C. pyruviciproducens* (200 μg/ml) or CP-PGN (50 μg/ml) for 3 hours. Total RNAs were isolated with Trizol (Invitrogen, Carlsbad, CA, USA) from cells according to the manufacture’s protocol. Total RNA (0.5 μg) was reverse transcribed into cDNA in 10 μl of reverse transcriptase reaction mixture using a RT-PCR reagent kit (Takara, Dalian, China). Quantitative real-time PCR technique (qRT-PCR) was performed on LightCycler480 (Roche Diagnostics, Indianapolis, IN) detection system using a SYBR Green1 qRT-PCR kit (Takara, Dalian, China). Gene expression was normalized against the reference gene β-actin. For PCR array analysis, relative gene expression levels were calculated compared with the average value of control group. The fowllowing factors were detected including TNF-α, IL-12, IL-10, iNOS and arginase-1.

### Flow cytometric analysis

The RAW264.7 cells and peritoneal macrophages (1 × 10^6^/well) were stimulated by heat-inactivated CP (200 μg/ml) or CP-PGN (50 μg/ml) for 24 hours, cell subpopulation markers CD86 of M1 macrophages was analyzed by flow cytometry to evaluate the different phenotypes. The cells were collected and washed with PBS. Cells were stained with PE-conjugated anti-CD86 (0.3 μg/1 × 10^6^ cells, Miltenyi Biotec) for 30 min in the dark. After washing, the expression levels of CD86 of cells were analyzed with a CyAn ADP 9 C flow cytometer (Beckman Coulter). Parallel sets of cells were incubated with monoclonal immunoglobulin isotype control antibody (Miltenyi Biotec) and the fluorescence intensity of these cells served as non-specific negative control. Data were analyzed using FlowJo software (TreeStar, Ashland, OR).

### Activation and differentiation of macrophages by CP and PGN *in vivo*

CP (500 μg/mouse) or CP-PGN (100 μg/mouse) were injected into the abdominal cavity of female BALB/c mice (age 6 to 8 weeks) with sterile PBS treated mice as control. After 24 hours peritoneal macrophages were collected to detect the level of NO by Griess assay and expresstion of cytokines by qRT-PCR. Furthermore, iNOS and arginase-1 were determined by qRT-PCR, and CD86 was measured by Flow cytometric analysis. All the test methods were similar to the description above and it’s just that the collected cells did not need to be stimulated again.

### Electrotransfection of siRNA

Small interfering RNA (siRNA) targeting mouse TLR2 and a control siRNA were obtained from GenePharma (Suzhou, China). Each transfection was performed with 2.5 × 10^6^/well. The cells were washed three times with PBS, then the cell solution was transferred to 4 mm electroporation cuvettes with 500 μl of pulsing buffer (RPMI1640) containing either 2 μl of siRNA of TLR2 or a control siRNA, the cuvettes were left to equilibrate at room temperature for 10 min before electroporation. One electric pulse was applied to the cuvettes at a field strength of 340 V/4 mm, 15 ms duration using a Bio-Rad Gene Pulser electroporation apparatus with capacitance extender. After pulsing, the cuvettes were kept on ice for 10 min to allow more siRNA enter to the cells, then plated them out to a 12-well plate with cell culture medium and incubated for 24 h after transfection, the inhibition ratio was detected by qRT-PCR. Meanwhile, 24 h after transfection, new medium was added with heat-inactivated CP or CP-PGN for 3 h or 24 h to perform the qRT-PCR, Griess assay, cell migration assay and flow cytometry analysis as described above.

### Statistical analysis

All data were shown as means ± Standard Error of Mean (SEM). Differences between two groups were compared by using the Student’s t-test. Survival analysis between three groups were done using Log-Rank test. All statistical analyses were performed by GraphPad Prism 5.0 (GraphPad Software, San Diego, CA). A *P*-values less than 0.05 was considered statistically significant.
